# A Noninvasive Comparison Study between Human Gliomas with IDH1 and IDH2 Mutations by MR Spectroscopy

**DOI:** 10.3390/metabo9020035

**Published:** 2019-02-20

**Authors:** Xin Shen, Natalie L. Voets, Sarah J. Larkin, Nick de Pennington, Puneet Plaha, Richard Stacey, James S. O. McCullagh, Christopher J. Schofield, Stuart Clare, Peter Jezzard, Tom Cadoux-Hudson, Olaf Ansorge, Uzay E. Emir

**Affiliations:** 1 Weldon School of Biomedical Engineering, Purdue University, West Lafayette, IN 47907, USA; shen363@purdue.edu; 2 Wellcome Centre for Integrative Neuroimaging, FMRIB Division, Nuffield Department of Clinical Neurosciences, University of Oxford, Oxford OX3 9DU, UK; natalie.voets@ndcn.ox.ac.uk (N.L.V.); stuart.clare@ndcn.ox.ac.uk (S.C.); peter.jezzard@univ.ox.ac.uk (P.J.); 3 Nuffield Department of Clinical Neurosciences, University of Oxford, Oxford OX3 9DU, UK; sarah.larkin@ocdem.ox.ac.uk (S.J.L.); Nicholas.DePennington@ouh.nhs.uk (N.d.P.); olaf.ansorge@ndcn.ox.ac.uk (O.A.); 4 Department of Neurosurgery, John Radcliffe Hospital, Oxford University Hospitals NHS Trust, Oxford OX3 9DU, UK; Puneet.Plaha@ouh.nhs.uk (P.P.); Richard.Stacey@ouh.nhs.uk (R.S.); Tom.Cadoux-Hudson@ouh.nhs.uk (T.C.-H.); 5 Chemistry Research Laboratory, Department of Chemistry, University of Oxford, Oxford OX1 3TA, UK; james.mccullagh@chem.ox.ac.uk (J.S.O.M.); christopher.schofield@chem.ox.ac.uk (C.J.S.); 6 School of Health Sciences, Purdue University, West Lafayette, IN 47907, USA

**Keywords:** glioma, isocitrate dehydrogenase, MR spectroscopy, metabolism, neurochemical profile

## Abstract

The oncogenes that are expressed in gliomas reprogram particular pathways of glucose, amino acids, and fatty acid metabolism. Mutations in isocitrate dehydrogenase genes (IDH1/2) in diffuse gliomas are associated with abnormally high levels of 2-hydroxyglutarate (2-HG) levels. The aim of this study was to determine whether metabolic reprogramming associated with IDH mutant gliomas leads to additional ^1^H MRS-detectable differences between IDH1 and IDH2 mutations, and to identify metabolites correlated with 2-HG. A total of 21 glioma patients (age= 37 ± 11, 13 males) were recruited for magnetic resonance spectroscopy (MRS) using semi-localization by adiabatic selective refocusing pulse sequence at an ultra-high-field (7T). For 20 patients, the tumor mutation subtype was confirmed by immunohistochemistry and DNA sequencing. LCModel analysis was applied for metabolite quantification. A two-sample t-test was used for metabolite comparisons between IDH1 (*n* = 15) and IDH2 (*n* = 5) mutant gliomas. The Pearson correlation coefficients between 2-HG and associated metabolites were calculated. A Bonferroni correction was applied for multiple comparison. IDH2 mutant gliomas have a higher level of 2-HG/tCho (total choline=phosphocholine+glycerylphosphorylcholine) (2.48 ± 1.01vs.0.72 ± 0.38, *P_c_* < 0.001) and myo-Inositol/tCho (2.70 ± 0.90 vs. 1.46 ± 0.51, *P_c_* = 0.011) compared to IDH1 mutation gliomas. Associated metabolites, myo-Inositol and glucose+taurine were correlated with 2-HG levels. These results show the improved characterization of the metabolic pathways in IDH1 and IDH2 gliomas for precision medicine.

## 1. Introduction

**Figure 1:**
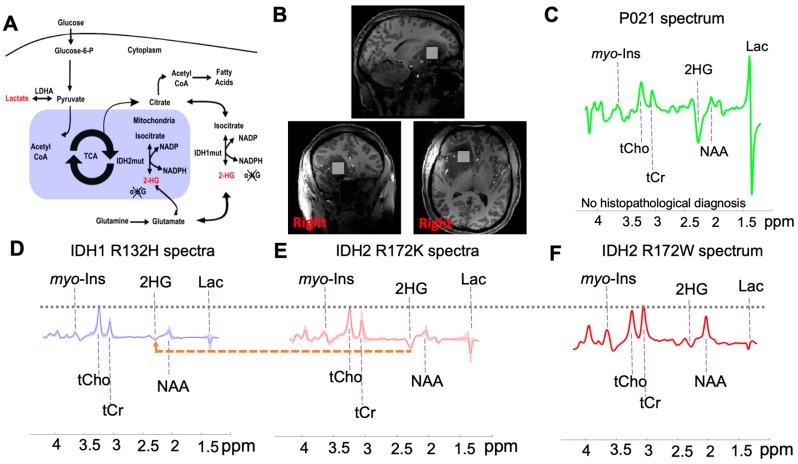
(**A**) Illustration of isocitrate dehydrogenase (IDH) function change with IDH1 (cytosol) and IDH2 (mitochondria) mutations, (**B**) representative tumor tissue voxel placement (P021), (**C**) individual spectrum from a glioma patient without histopathological diagnosis, (**D**) mean ± standard deviation (SD) of the spectra from fifteen IDH1 R132H mutant patients, (**E**) mean ± SD of the spectra from four IDH2 R172K mutant patients, and (**F**) individual spectrum from an IDH2 R172W mutant patient. The vertical scale was normalized to the total choline signal (tCho = phosphocholine + glycerylphosphorylcholine). In addition, the peaks of 2-hydroxyglutarate (2-HG), tCho, total creatine (tCr = creatine + phosphocreatine), N-acetylaspartate (NAA), myo-inositol (myo-Ins), and lactate (Lac) were highlighted in Figure (**B**–**E**).

In the post-genomics era, the World Health Organization (WHO) classification of gliomas has become even more tightly integrated with molecular parameters in addition to histology [1]. Integrated diagnoses offer prospects for precision medicine strategies tailoring therapies for each individual, by delivering more effective treatments, whilst avoiding or reducing adverse outcomes. Mutations in the genes encoding for isocitrate dehydrogenase (IDH) have been discovered in various cancers such as glioma, acute myeloid leukemia (AML), chondrosarcoma, cholangiocarcinoma, paraganglioma, colon cancer, prostate cancer and lung cancer [2]. Isocitrate dehydrogenase is an intracellular enzyme, that catalyzes the reversible production of alpha-ketoglutarate (α-KG) from isocitrate in the cytosol (IDH1) and mitochondria (IDH2) as seen in Figure 1A [2]. The IDH mutation, which is present in about 80% of so-called “low-grade” (WHO grade II) gliomas and secondary glioblastomas, plays an important role in tumorigenesis [3] and results in a loss of enzyme function, production of 2-hydroxyglutarate (2-HG), and likely consequent DNA cytosine-hypermethylation [4]. Over 90% of the reported IDH mutations in glioma patients affects the IDH1 gene at codon R132H, whereas mutations in the IDH2 gene are less common, affecting 2.4% of gliomas [5]. The frequency of IDH2 R172K mutations in IDH-mutated glioma is 1.4%, followed by R172M (0.6%) and R172W (0.2%) [5].

The clinical practice for glioma patients is becoming increasingly personalized by subdividing patients into groups based on molecular tumor characteristics. For instance, IDH mutant gliomas are more sensitive to treatment-induced oxidative cellular damage, suggesting different treatment paradigms for mutant and wild-type gliomas [6]. Specifically, IDH2 R172 mutation has a poorer overall survival following the treatment [7,8,9]. Thus, it is becoming clear that diagnostic imaging tools, in particularly magnetic resonance imaging (MRI), may play a critical role in precision medicine strategies for glioma patients including early diagnosis, guiding treatment, and evaluating response to therapies [10]. Although immunohistochemical (IHC) and molecular pathological analysis of surgically obtained tumor tissue is still the gold standard for the diagnosis of an IDH mutated glioma [11,12], in vivo diagnosis of IDH mutant glioma patients has been demonstrated by utilizing proton magnetic resonance spectroscopy (^1^H MRS) to detect 2-HG [13,14,15,16]. In recent times, ^1^H MRS has also been used for monitoring the treatment response of adjuvant radiation and chemotherapy [17] and investigational drugs [18]. Ultra-high-field (UHF, ≥7T) MRI scanners offer enhanced detection, relative to routine 3T MRI, of 2-HG peaks in the magnetic resonance spectroscopy (MRS) spectra of IDH mutated patients. Furthermore, at 7T, this 2-HG peak is a visually discernible signal, detectable with as little as 5–10 s of data acquisition, suggesting sufficient sensitivity, not only to detect subtle changes due to the reprogramming of cellular metabolism during the disease progression or treatment, but also to discriminate between common IDH1 and rare IDH2 mutations [16,19].

The metabolic reprogramming associated with IDH mutation, leads to alternations in cellular metabolism beyond 2-HG production [20]. Further investigation of how 2-HG production impacts the other metabolic pathways might provide additional insights into their metabolic reprogramming. The in vivo detection of other metabolites, and their up/down regulation relative to 2-HG production, might offer diagnostic or prognostic value for tumor subtyping/grading and monitoring the response to treatments. Several efforts have identified selective pharmacological agents that target IDH1 and IDH2 mutations [21]. However, importantly, it has been reported that the decrease in 2-HG levels was not solely a predictive marker of the treatment response in an inhibitor of the mutant IDH2 drug trial of acute myeloid leukemia [22]. Thus, the relationship between 2-HG and associated metabolites may provide useful information on the assessment of the pharmacodynamics, efficiency, and the toxicity, of these treatments in glioma patients.

In recent years, non-invasive detection of 2-HG in IDH mutant gliomas has emerged as one of the most significant advances in the field of non-invasive diagnostic imaging for precision medicine [23]. The objective of our study was to demonstrate the potential of ^1^H MRS to distinguish neurochemical profile differences between the IDH1 and IDH2 mutations at UHF MRI. Our results suggest that ^1^H MRS could be useful as a prognostic precision medicine biomarker detection system for identifying, stratifying, and monitoring IDH1 and IDH2 mutant glioma patients.

## 2. Results

### 2.1. Study Cohort

Patient demographics and in vivo MRS-derived 2-HG concentrations are summarized in Table 1. Figure 1 shows representative spectra of tumor voxels from the IDH1 R132H group (Figure 1D), the IDH2 R172K group (Figure 1E), and two individual patients (Figure 1F shows a patient with an IDH2 R172W mutation; Figure 1C depicts one patient without any histopathological diagnosis) obtained at 7T. The localization accomplished by semi-localization by adiabatic selective refocusing (semi-LASER) and outer volume suppression (OVS) eliminated signals from outside the volume of interest (VOI) and resulted in artifact-free spectra with a flat baseline in the spectral range [0.5, 4.2] ppm. The choice of echo time (110 ms) resulted in an inverted and visually discernable peak at 2.25 ppm, as previous described in Emir et al. [19]. Due to the chemical shift dispersion at UHF, the overlap of the H4–H4’ resonance of 2-HG (at 2.25 ppm) with neighboring metabolites was minimized [19]. IDH2 R172K and R172W (Figure 1E,F) mutations lead to higher levels of 2-HG compared with IDH1 R132H (Figure 1D).

### 2.2. Subtyping IDH Mutant Gliomas

**Figure 2:**
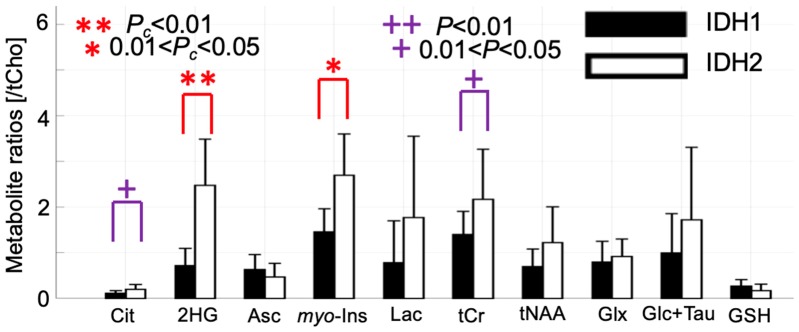
Metabolite ratios of citrate (Cit), 2-hydroxyglutarate (2-HG), ascorbate (Asc), myo-Inositol (myo-Ins), lactate (Lac), total creatine (tCr = creatine + phosphocreatine), total N-acetylaspartate (tNAA = N-acetylaspartate + N-acetylaspartylglutamate), Glx (= Gln + Glu), glucose+ taurine (Glc+Tau), and glutathione (GSH) over total choline (tCho = phosphocholine + glycerylphosphorylcholine) in IDH1 and IDH2 mutant gliomas. The metabolite ratios showing a significant difference between IDH1 and IDH2 mutations are highlighted by red star(s) (after Bonferroni correction for multiple comparison) and purple plus sign(s) (before Bonferroni correction).

Quantification of 2-HG and associated metabolite concentrations using LCModel are illustrated in Figure 2. The high spectral quality enabled the quantification of a neurochemical profile consisting of 10 metabolites (Figure 2). In line with the spectral pattern observed in Figure 1D–F, the metabolite ratio of 2-HG/tCho (tCho = phosphocholine + glycerylphosphorylcholine) signal was higher in the IDH2 mutation samples compared to the IDH1 samples (2.48 ± 1.01 vs. 0.72 ± 0.38, *P_c_* < 0.001) (*P_c_* Bonferroni corrected *p*-values and *P* uncorrected *p*-values). In addition, IDH2 mutation samples manifest a higher myo-Inositol/tCho (2.70 ± 0.90 vs. 1.46 ± 0.51, *P_c_* = 0.011), and a trend to increase (significant *p*-values which did not survive after Bonferroni correction) in citrate/tCho (0.20 ± 0.11 vs. 0.11 ± 0.06, *P* = 0.034), total creatine/tCho (2.17 ± 1.10 vs. 1.40 ± 0.51, *P* = 0.043).

**Figure 3:**
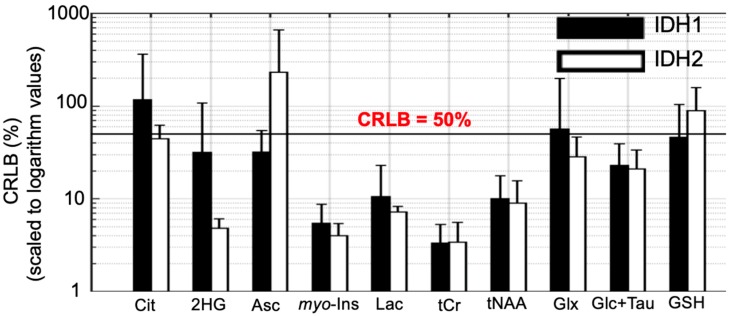
Cramer-Rao lower bound (CRLB) of citrate (Cit), 2-hydroxyglutarate (2-HG), ascorbate (Asc), myo-inositol (myo-Ins), lactate (Lac), total creatine (tCr = creatine + phosphocreatine), total N-acetylaspartate (tNAA = N-acetylaspartate + N-acetylaspartylglutamate), Glx (= glutamine + glutamate), glucose+ taurine (Glc+Tau), and glutathione (GSH) in IDH1 and IDH2 mutant gliomas. The vertical axis is scaled to logarithm values. CRLBs of IDH2 patients did not differ from those of IDH1 patients.

The mean Cramer-Rao lower bounds (CRLBs), the goodness of the fit, of metabolite fitting are compared across IDH-mutated tumor spectra in Figure 3. Due to the higher 2-HG signal in IDH2 mutations compared to IDH1 mutations, the CRLB for 2-HG resulted in lower values in IDH2 mutations (31.8% ± 76.2% vs. 4.8% ± 1.3%). Similarly, citrate/tCho (117.6% ± 245.2% vs. 44.6% ± 17.6%), and lactate/tCho (10.5% ± 12.5% vs. 7.2% ± 1.1%) showed reduced CRLBs for IDH2 relative to IDH1.

### 2.3. 2-HG and Associated Metabolites Concentrations

**Figure 4:**
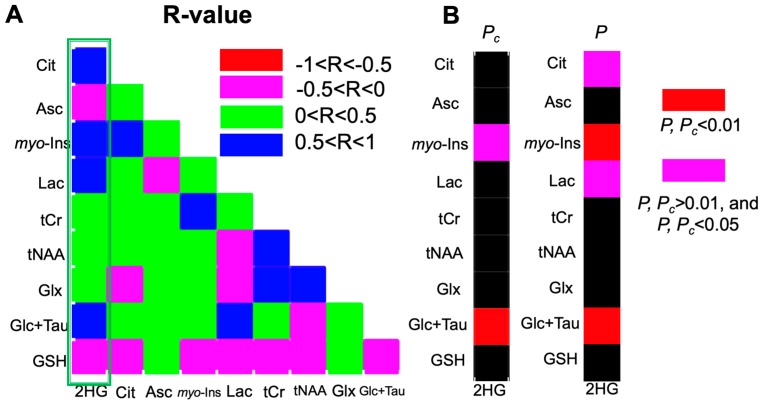
(**A**) Heat map of Pearson correlation coefficients (r) between each pair of citrate (Cit), 2-hydroxyglutarate (2-HG), ascorbate (Asc), myo-Inositol (myo-Ins), lactate (Lac), total creatine (tCr = creatine + phosphocreatine), total N-acetylaspartate (tNAA = N-acetylaspartate + N-acetylaspartylglutamate), Glx (= glutamine + glutamate), glucose+ taurine (Glc+Tau), and glutathione (GSH) from all IDH mutant patients. (**B**) Heat maps of *P_c_* (Bonferroni corrected *p*-values) and *P* (uncorrected *p*-values) between 2-HG and associated metabolites. The significant correlation was highlighted by red color (*P_c_* or *P* < 0.01) and magenta color (*P_c_* or *P* < 0.05, but *P_c_* or *P* > 0.01).

**Figure 5:**
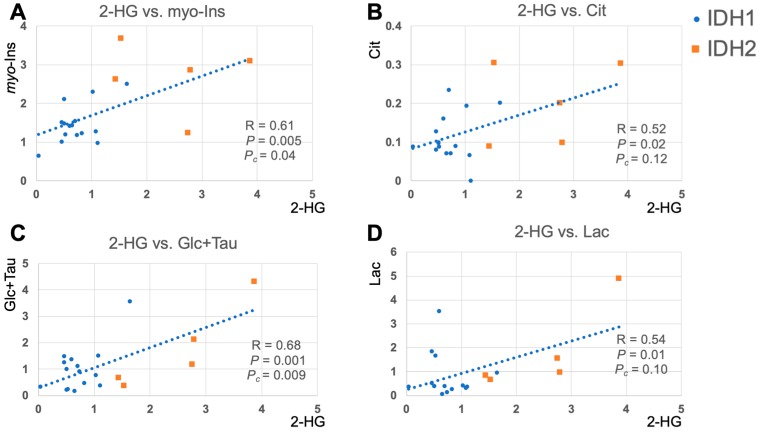
Scatter plots of 2-hydroxyglutarate (2-HG) and significantly correlated metabolites. (**A**) 2-HG and myo-Inositol (myo-Ins). (**B**) 2-HG and citrate (Cit). (**C**) 2-HG and glucose+ taurine (Glc+Tau). (**D**) 2-HG and lactate (Lac). *P*: uncorrected *p*-values. *P_c_*: Bonferroni corrected *p*-values. IDH1 patients were shown in blue dots, and IDH2 patients were shown in orange squares. The linear trendline was calculated based on all IDH patients.

The results of correlation analyses for all IDH patients between 2-HG and other metabolites are illustrated in Figure 4 and Figure 5. This analysis indicated a positive correlation between 2-HG, and myo-Inositol (R = 0.61, *P_c_* = 0.04), glucose + taurine (R = 0.68, *P_c_* < 0.01), as well as trends of positive correlation between 2-HG and citrate (R = 0.52, *P* = 0.019), lactate (R = 0.54, *P* = 0.014) signals. 

## 3. Discussion

To our knowledge, this is the first in vivo report investigating the metabolic differences between glioma patients harboring IDH1 and IDH2 mutations. The results reveal that an optimized semi-LASER MRS sequence at UHF offers high sensitivity and localization for the in vivo detection of 2-HG, characteristic of IDH mutated brain tumors. The improved spectral dispersion at UHF was found to result in the detection of up to ten additional metabolites. In addition to the further increase in the ratio of 2-HG/tCho, we found a significantly higher metabolite ratio of myo-Inositol/tCho in IDH2 gliomas (four patients with IDH2 R172K and one patient with IDH2 R172W mutations) compared to IDH1 gliomas (fifteen patients with IDH1 R132H mutation). The correlation analysis revealed that the 2-HG/tCho ratio correlated with several metabolite ratios, i.e., for myo-Inositol/tCho, and glucose+taurine/tCho.

Compared to our previous studies [16,19], nine additional histopathologically confirmed IDH mutant glioma patients were recruited in this follow-up study, as well as one more patient showing a visually discernible 2-HG peak at 2.25 ppm but who declined surgery and therefore remains without histopathological diagnosis. The excellent spectral quality obtained at 7T enabled us to detect elevated 2-HG concentrations in glioma patients harboring common (i.e., IDH1 R132H) and rare IDH mutations (i.e., IDH2 R172K and R172W). Owing to the relatively high frequency of IDH2 mutations in our study cohort (20%), we were able to compare the neurochemical profiles between IDH1 and IDH2 mutations further. First of all, in accord with in vitro studies [24,25], the mitochondrial IDH2 mutations manifested a significantly elevated 2-HG/tCho metabolite ratio compared to gliomas with the cytosolic IDH1 mutation. Furthermore, our data indicates that the myo-Inositol/tCho metabolite ratio is higher in mitochondrial IDH2 gliomas compared to cytosolic IDH1 gliomas. This observation is consistent with an in vitro study showing a 20% increase in myo-Inositol level in IDH2 compared to IDH1 [26]. A recent in vivo survey supports this by demonstrating a link between myo-Inositol concentration and hypermethylation in the expression of inositol 3-phosphate synthase (ISYNA1) in brain tumors [27]. Another recent study of T-cell lymphoma has also reported a significantly increased hypermethylation in ISYNA1 for IDH2 mutant samples [28].

Our data also indicates a trend for an increased citrate/tCho metabolite ratio in gliomas carrying an IDH2 mutation, in line with a previous study by Reitman et al. [26]. Together with the elevation in glucose+taurine/tCho (1.72 ± 1.59 vs. 0.99 ± 0.86) and lactate/tCho (1.77 ± 1.78 vs. 0.79 ± 0.0.92) metabolite ratios, these observations suggest that IDH2 mutated gliomas might favor oxidative phosphorylation over aerobic glycolysis compared to IDH1 due to metabolic reprogramming associated with IDH mutation. This proposal is supported by a recent study demonstrating enriched gene sets related to oxidative phosphorylation in the IDH2 mutation subset of glioma patients [29].

The excess production of 2-HG or reduced α-KG in IDH mutations (Figure 1A) suggests that 2-HG can be directly involved in promoting tumorigenesis [3]. However, recent studies have found that it would be insufficient to predict the clinical response of IDH mutant patients by detecting 2-HG concentration alone [22]. Given that our study cohort was solely IDH-mutant, this provided a unique opportunity to investigate this phenomenon via a metabolomics approach, since correlations of 2-HG levels with other associated metabolites might provide additional insight on the cancer-promoting activity of 2-HG. In this study, we found that metabolites including myo-Inositol and glucose+taurine were positively correlated with 2-HG, which is the oncometabolite of IDH mutations [30]. In addition, we found trends of a positive correlation between 2-HG and citrate, lactate, as well as a trend for a negative correlation between 2-HG and glutathione concentrations, which is in-line with previous in vivo MRS and cell line studies [15,31]. These results demonstrate that, analyzing in vivo UHF ^1^H MRS data via metabolomics approaches can effectively complement conventional methods, not only by revealing insights into the underlying metabolic pathways of tumorigenesis, but also for monitoring the pharmacodynamics and the identification of new pharmacological targets of anti-cancer treatments.

This is a small study, particularly in regard to patients with rare IDH2 mutations, however, the metabolite ratio distinction between the canonical IDH1 R132H and IDH2 mutations is robust. Another limitation of this study is the reporting of metabolite ratios using an internal tCho reference within tumor voxels. In previous work, since there was no observed difference in the 2-HG/tCho metabolite ratio measured at 3T and 7T, compared to metabolite ratios using an internal total creatine (= creatine + phosphocreatine) [16,19], we chose to report metabolite ratios by referencing to tCho due to the practicality of translating our findings to 3T.

## 4. Materials and Methods

Written and informed consent was obtained from every participant in the study, which was approved by the ethics committee.

### 4.1. Study Cohort

A total of 21 patients (age = 37 ± 11, 13 males) with a pre-operative radiological diagnosis of a glioma were recruited for a 7T MRI scan using a whole-body MR system (Siemens, Erlangen, Germany) with a Nova Medical single-channel transmit and 32-channel receive array head-coil. The mutation subtype (IDH1 or IDH2) was assessed by IHC and DNA sequencing from a surgical tissue biopsy [16,19]. Following surgical treatment, fifteen patients (age = 41 ± 10, 12 males) were identified as having an IDH1 mutation, while five patients (age = 27 ± 5, 4 females) were found to carry an IDH2 mutation. No histopathological diagnosis could be established for one glioma patient (P021), and this patient was excluded from statistical analysis. The demographic parameters are summarized in Table 1. A subset of the data has previously appeared in published work at 3T [16] and 7T [19].

### 4.2. Data Acquisition

A T1-weighted anatomical MPRAGE scan was acquired in all patients at the beginning of the scan to identify a region of interest for MRS measurements within the tumor. The MPRAGE sequence parameters were: resolution = 1 × 1 × 1 mm^3^, repetition time (TR) = 2.3 s, echo time (TE) = 2.8 ms, inversion time (TI) = 1.05 s, and total scan time = 3 min. Prior to MRS acquisitions, first-order and second-order shims were applied via a gradient-echo shim mapping, and then using a FASTMAP method only for the fine adjustment of the first-order shim [32]. In addition, in order to increase the extent of the effective transmit field (B_1_^+^), external barium titanate pads were placed over the expected location of the tumor [33]. After these preparations, a VOI was selected within the tumor region, and a semi-LASER pulse sequence was applied to each VOI for spectra measurements [34]. The 2-HG multiplets at 2.25 ppm (H4, H4’) have lead to a maximum absorptive negative (inverted) multiplet at a total echo time of 100–120 ms. A TE of 110 ms was chosen, since a near fully absorptive negative 2-HG and lactate (Lac) spectral pattern at 2.25 ppm and 1.35 ppm with timings TE_1_= 11 ms, TE_2_ = 65 ms and TE_3_ = 34 ms (total TE = 110 ms). The spectroscopy acquisition parameters were as below: volume size = 20 × 20 × 20 mm^3^ = 8 mL (except one subject with a volume size = 20 × 10 × 10 = 2 mL), TE = 110 ms, TR = 5–6 s, number of transients NT = 128, spectral bandwidth = 6 kHz, and data points = 2048. In addition, water suppression was done by variable power and optimized relaxation (VAPOR) delays, and OVS was applied with an 8 mm distance between the VOI edge and each OVS saturation band to ensure no signal loss within the VOI. Within the same VOI, one non-suppressed water spectrum, with the VAPOR scheme turned off, was acquired for eddy current correction.

### 4.3. Simulations

Spectra were phase and frequency corrected and fitted with LCModel using simulated basis sets [19] containing 22 metabolites: citrate (Cit), 2-hydroxyglutarate (2-HG), alanine (Ala), ascorbate (Asc), aspartate (Asp), creatine (Cr), gamma-aminoburytic acid (GABA), glutamine (Gln), glutamate (Glu), glycine (Gly), myo-inositol (myo-Ins), lactate (Lac), N-acetylaspartate (NAA), N-acetylaspartylglutamate (NAAG), phosphocholine (PCho), phosphocreatine (PCr), phosphorylethanolamine (PE), scyllo-inositol (sIns), taurine (Tau), glucose (Glc), glycerylphosphorylcholine (GPC) and glutathione (GSH). Concentrations are reported relative to an internal reference of total choline (tCho = PCho + GPC) (Figure S1). No correction for *T*_2_ decay differences between metabolites was carried out in this work. It has been shown that CRLB based exclusion criteria for poor quality spectra might lead to wrong or missed statistical findings [35]. In this study, we therefore chose to analyze data without quality filtering based on CRLB percentages in order to eliminate such potential bias. Thus, metabolites were included in statistical analysis only if the CRLB was less than 99% in more than half of the spectra, and no additional threshold was applied to exclude any data within these metabolites. The summation of two metabolites were reported instead of single metabolites if the correlation between these two was consistently high (correlation coefficient < −0.5). In summary, the metabolite ratios of Cit/tCho, 2-HG/tCho, Asc/tCho, myo-Ins/tCho, Lac/tCho, tCr (total creatine, = Cr + PCr)/tCho, tNAA (total NAA, = NAA + NAAG)/tCho, Glx (= Gln + Glu)/tCho, Glc+Tau/tCho, and GSH/tCho were reported.

### 4.4. Statistical Analysis

A two-sample t-test was used to examine statistical differences in metabolite ratios between confirmed IDH1 and IDH2 mutations (a total of five patients; four patients with IDH2 R172K and one patient with IDH2 R172W mutations). In addition, Pearson correlation coefficients (R) were calculated between the LCModel output value of 2-HG/tCho and the ratio of associated metabolites over tCho. Bonferroni correction was applied for multiple comparison. For all analyses, a *p*-value of < 0.05 was considered to indicate statistical significance.

## 5. Conclusions

In summary, the high-quality spectra in this study enabled the quantification of neurochemical profiles consisting of at least ten metabolites, including 2-HG, glutamine+glutamate, lactate, and glutathione in both IDH1 and IDH2 mutations. This enabled us to demonstrate that the noninvasive measurement of metabolic reprogramming associated IDH mutant brain tumors via in vivo MRS holds excellent value for a molecule-specific, clinically relevant, personalized biomarker. The analysis of 2-HG and associated metabolites with larger sample sizes can potentially provide metabolic characteristics which can be used for tumor classification, survival prediction, treatment planning and monitoring of therapy and post-therapy evaluation. Our results suggest that UHF ^1^H MRS could be useful as a prognostic precision medicine biomarker detection system for identifying, stratifying, and monitoring IDH1 and IDH2 mutant glioma patients towards the goal of precision medicine of gliomas.

## Figures and Tables

**Table 1:** Demographic and clinical characteristics of glioma patients.

Subject ID	Age, Gender	Prior Oncological Treatment	Diagnosis	IHC	DNA Sequencing	2HG/tCho	2HG CRLB
P001	29, M	None	Astrocytoma (WHO grade 2)	+ve	N/A	0.60	20
P002	30, M	None	Anaplastic astrocytoma (WHO grade 3)	+ve	N/A	0.70	8
P003	35, F	Radiotherapy	Anaplastic oligoastrocytoma (WHO grade 3)	+ve	N/A	0.46	30
P004	34, M	None	Anaplastic astrocytoma (WHO grade 3)	+ve	N/A	1.64	7
P005	47, M	None	Anaplastic astrocytoma (WHO grade 3)	+ve	N/A	0.46	20
P006	57, F	None	Astrocytoma (WHO grade 2)	+ve	N/A	0.73	9
P007	51, M	None	Anaplastic astrocytoma (WHO grade 3)	+ve	N/A	0.51	9
P008	48, M	None	Anaplastic oligodendroglioma (WHO grade 3)	+ve	N/A	0.52	21
P009	53, M	None	Anaplastic astrocytoma (WHO grade 3)	+ve	N/A	0.82	6
P010	45, M	None	Oligodendroglioma (WHO grade 2)	+ve	N/A	0.65	12
P011	44, F	None	Anaplastic astrocytoma (WHO grade 3)	+ve	N/A	0.50	13
P012	20, M	None	Astrocytoma (WHO grade 2)	+ve	N/A	0.04	306
P013	36, M	None	Anaplastic astrocytoma (WHO grade 3)	+ve	N/A	1.10	4
P014	51, M	None	Oligodendroglioma (WHO grade 2)	+ve	N/A	1.08	5
P015	36, M	None	Astrocytoma (WHO grade 2)	+ve	N/A	1.02	7
P016	27, F	None	Oligodendroglioma (WHO grade 2)	-ve	IDH2 R172K	2.79	4
P017	29, F	None	Oligodendroglioma (WHO grade 2)	-ve	IDH2 R172K	2.76	3
P018	32, F	Chemotherapy	Oligodendroglioma (WHO grade 2)	-ve	IDH2 R172K	3.87	5
P019	19, F	None	Oligodendroglioma (WHO grade 2)	-ve	IDH2 R172W	1.54	6
P020	26, M	None	Anaplastic astrocytoma (WHO grade 3)	-ve	IDH2 R172K	1.44	6
P021	29, F	None	N/A	N/A	N/A	12.82	3

Abbreviations: +ve, immunopositive; -ve, immunonegative; N/A, not applicable; WHO, World Health Organization; CRLB, Cramer-Rao lower bound; 2-HG, 2-hydroxyglutarate; tCho, total Choline = phosphocholine + glycerylphosphorylcholine; IDH, isocitrate dehydrogenase.

## Supplementary Materials

The following are available online at http://www.mdpi.com/2218-1989/9/2/35/s1, Figure S1: LCModel analysis.
