# Supplementary material for: A Noninvasive Comparison Study between Human Gliomas with IDH1 and IDH2 Mutations by MR Spectroscopy
Source: Metabolites. 2019 Feb 20;9(2):35. doi: 10.3390/metabo9020035 (PMC6409728; doi:10.3390/metabo9020035)
Supplement: Supplementary file 1 [file metabolites-09-00035-s001.pdf]

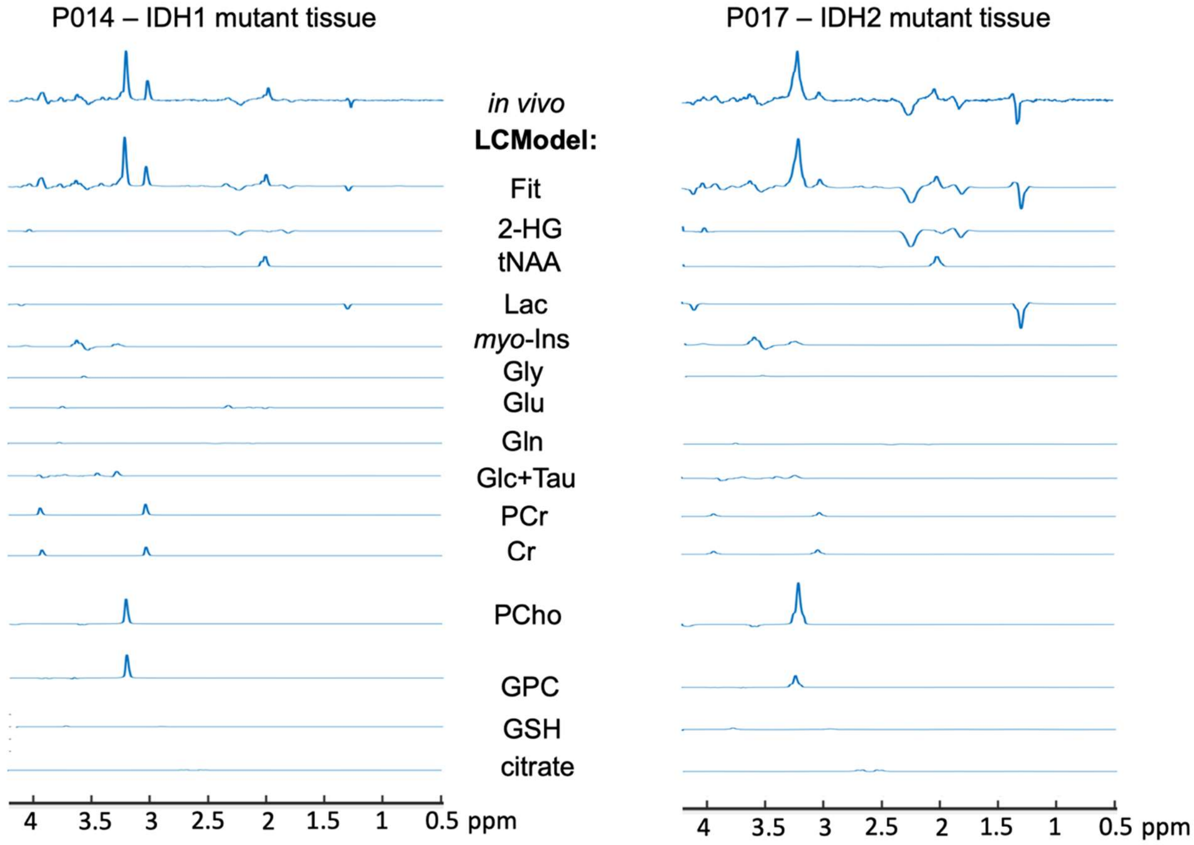

Figure S1: LCModel analysis. In vivo semi-LASER spectra from IDH1 and IDH2 patients (volume of interest, 8 ml, TE = 110 ms, TR = 5s and number of transients, 128) and LCModel fit, modelling metabolite contributions to the neurochemical profile. The vertical scale was normalized to the total choline signal. Model spectra for 2-hydroxyglutarate (2-HG), alanine (Ala), glycerophosphocholine (GPC), phosphocholine (PC), citrate, creatine (Cr), phosphocreatine (PCr), glucose+taurine (Glc+Tau), glutamine (Gln), Glutamate (Glu), glutathione (GSH), glycine (Gly), lactate (Lac), myo-inositol (myo-Ins) and total NAA (tNAA) generated by using GAMMA/PyGAMMA simulation library of VESPA were imported into LCModel and used for spectroscopic quantification.
